# Mechanical–enzymatic isolation and characterization of primary human gingival epithelial cells for reproducible in vitro oral mucosa models

**DOI:** 10.1007/s00418-025-02422-0

**Published:** 2025-10-08

**Authors:** Henry Bautista-Amorocho, Jorge Alexander Silva-Sayago

**Affiliations:** 1 Universidad del Cauca, Grupo de Investigación en Salud GIS, Facultad de Ciencias de La Salud, Popayán, Colombia; 2 Universidad de Santander, Grupo de Investigación en Manejo Clínico CliniUDES, Facultad de Ciencias Médicas y de La Salud, Bucaramanga, Colombia

**Keywords:** Gingiva, Keratinocytes, Cell culture techniques, Immunohistochemistry, Epithelial cells, Oral mucosa

## Abstract

The establishment of reliable in vitro oral mucosa models is essential for advancing studies in epithelial barrier function, wound healing, and host–microbe interactions. However, the widespread use of immortalized cell lines such as HaCaT or TR146 limits physiological relevance owing to altered differentiation profiles and genetic drift. In this study, we developed a robust mechanical–enzymatic protocol for isolating and expanding primary human gingival keratinocytes from healthy gingival explants without feeder layers. The resulting cells demonstrated high viability, maintained consistent proliferative capacity across passages, and exhibited characteristic cobblestone morphology. Comprehensive phenotypic validation included immunofluorescence and immunohistochemistry confirming strong expression of epithelial markers CK18, AE1/AE3, and MUC1, with absence of the mesenchymal marker vimentin. Transcriptomic analysis using RT-qPCR corroborated epithelial lineage fidelity, revealing stable MUC1 expression and lack of MUC5AC transcripts, indicative of a nonglandular phenotype. Metabolic competence was supported by WST-1 assays that correlated strongly with manual cell counts, underscoring functional viability. Importantly, AGS and 293T/17 cell lines were processed in parallel as orthogonal controls to confirm assay specificity and lineage discrimination. Under rigorously standardized, within-laboratory conditions, our workflow yielded high interdonor concordance in epithelial identity and growth kinetics across a young-adult cohort (*n* = 3), supporting its use as a practical primary-cell platform for downstream applications. Generalizable reproducibility—across age strata, operators, and sites—will require formal, preregistered multicenter validation. By mitigating limitations inherent to immortalized lines, this approach enables more accurate investigations of epithelial biology and strengthens the reliability of in vitro experimental systems relevant to oral regenerative medicine and mucosal immunology.

## Introduction

The oral mucosa forms a dynamic epithelial barrier that plays essential roles in immunity, tissue repair, and homeostasis. Its stratified squamous epithelium, primarily composed of keratinocytes, protects against mechanical stress, microbial colonization, and chemical exposure. Gingival keratinocytes are especially relevant owing to their constant interaction with the oral biofilm and their role in modulating inflammation and facilitating wound healing (Zhang et al. 2020; Lamont et al. 2018). Beyond serving as a physical shield, these cells participate in orchestrating a complex network of signaling pathways that regulate innate and adaptive immune responses, contributing to periodontal health and influencing systemic inflammation. The unique anatomical and functional characteristics of the gingival epithelium make it a critical site for investigating host–microbe interactions and regenerative approaches.

In vitro models using oral keratinocytes are indispensable for studying epithelial stratification, host–pathogen interactions, and mucosal regeneration. Immortalized lines such as HaCaT and OKF6 offer scalability advantages but often deviate from in vivo phenotypes owing to altered differentiation, signaling, and gene expression (Colley et al. 2013). These deviations can compromise the reliability of mechanistic studies, particularly when assessing barrier function, cytokine responses, or interactions with biomaterials. In contrast, primary gingival keratinocytes better retain native epithelial features and immune responsiveness, providing more physiologically relevant models (Odioso et al. 1995; Zhang et al. 2022). They also enable personalized investigations reflecting individual variability in inflammatory profiles and wound healing capacity, which are essential for translational applications in precision medicine.

Isolation techniques include explant-based and enzymatic protocols. Explant cultures preserve tissue architecture and reduce enzymatic damage, but yield fewer cells and require longer culture times (Zhang et al. 2022; Gasiorek et al. 2021). Enzymatic methods provide higher efficiency but may risk compromising junctional integrity. When performed under serum-free, feeder-free conditions, enzymatic dissociation improves standardization and between-sample consistency across donor-derived cultures (Zhang et al. 2020; Nakamura et al. 2016). Integrating mechanical steps with enzymatic digestion can further enhance cell yield while minimizing potential damage to critical surface proteins and intercellular junctions, facilitating the generation of high-quality epithelial sheets suitable for downstream applications.

Reliable epithelial models are critical to investigate microbial invasion, immune signaling, and barrier remodeling. *Porphyromonas gingivalis*, for example, modulates epithelial migration and transcription under homeostatic and pathological conditions (Lamont et al. 2018; Sztukowska et al. 2016). Primary gingival keratinocytes are widely used in two-dimensional (2D) and three-dimensional (3D) culture systems to study host defense, epithelial plasticity, and mucosal inflammation (Zhang et al. 2022; Gasiorek et al. 2021). These models serve as valuable platforms for evaluating novel therapeutic agents, testing bioactive materials, and elucidating molecular mechanisms involved in oral disease pathogenesis. Furthermore, the use of well-characterized primary keratinocytes supports the development of bioengineered grafts and mucosal constructs, potentially improving clinical outcomes in periodontal and peri-implant regenerative therapies.

This study aimed to develop and validate a standardized, robust protocol for mechanical–enzymatic isolation of human gingival keratinocytes. Cultures were evaluated for morphology, proliferative behavior, and epithelial marker expression to establish a robust and fibroblast-free in vitro model applicable to periodontal research, epithelial biology, and tissue engineering. In contrast to previously published models, this protocol uniquely integrates donor-derived monolayers with strict epithelial validation, offering a scalable and phenotypically faithful platform for translational applications, enabling future mechanistic studies on barrier function, microbial challenges, and wound healing in patient-derived models.

## Materials and methods

### Study design

This research was conceived as an in vitro experimental study specifically aimed at establishing and validating a standardized protocol with demonstrated interdonor consistency for the mechanical–enzymatic isolation and detailed characterization of primary gingival epithelial cells (GECs) derived from healthy human donors. The study design was structured to ensure consistency across different donors, minimize technical variability, and provide robust data for translational research applications in regenerative dentistry and oral epithelial biology.

### Human oral tissue samples

Gingival biopsies were obtained from three systemically healthy adult donors undergoing elective esthetic gingivectomy procedures in a controlled clinical setting. Inclusion criteria were: (i) age 18, 26, and 30 years (one donor each); (ii) absence of visible morphological alterations of the oral mucosa; (iii) no evidence of active soft or hard tissue infections; and (iv) absence of clinical or radiographic signs of gingivitis or periodontitis. Immediately after surgical excision, samples were carefully placed on ice in sterile tubes containing transport medium and transported under cold conditions to the cell culture laboratory. All specimens were processed within a maximum of 2 h post-extraction to ensure optimal preservation of cell viability and tissue integrity.

### Preparation of media

The transport medium consisted of high-glucose Dulbecco’s modified Eagle’s medium (DMEM; Gibco, Thermo Fisher Scientific, Waltham, MA, USA), supplemented with penicillin (200 U/mL), streptomycin (200 µg/mL), gentamicin (50 µg/mL), and amphotericin B (5 µg/mL), all sourced from Gibco.

The digestion medium was prepared using Hank’s balanced salt solution (HBSS) containing Ca^2+^/Mg^2+^ (Caisson, Smithfield, UT, USA) and included collagenase type I (200 U/mL; Sigma-Aldrich, St. Louis, MO, USA), dispase II (1.2 U/mL; Sigma-Aldrich), soybean trypsin inhibitor (0.01 mg/mL; ChemCruz, Dallas, TX, USA), bovine serum albumin (1.25 mg/mL; Caisson), and dithiothreitol (0.1 mM; PanReac AppliChem, Barcelona, Spain). This solution was sterile-filtered through 0.22 µm filters and stored at −80 °C in aliquots of 10 mL. Heat-labile supplements were added fresh on the day of use.

The proliferation medium was based on William’s E medium (Caisson), supplemented with 20% fetal bovine serum (FBS; Cell Applications, San Diego, CA, USA), l-glutamine (25 mM; Caisson), 4-(2-hydroxyethyl)-1-piperazineethanesulfonic acid (HEPES) buffer (10 mM; Caisson), recombinant human epidermal growth factor (EGF; 5 ng/mL; Cell Applications), insulin–transferrin–selenium (ITS; 1%; Thermo Fisher Scientific), and the same antibiotics as used in the transport medium. Transport and proliferation media were sterile-filtered and stored at 4 °C (≤ 4 weeks); the digestion medium was aliquoted and stored at −80 °C. Heat-labile supplements were added fresh on the day of use.

### Isolation and culture of primary gingival epithelial cells (GECs)

All tissue processing steps were performed aseptically in a class II biosafety cabinet (Thermo Scientific, Waltham, MA, USA). Biopsies were rinsed three times with precooled transport medium to eliminate blood residues and debris, then finely minced into approximately 1 mm^3^ fragments using sterile surgical scalpels. Fragments were transferred into 30 mL of digestion medium and incubated at 37 °C with continuous orbital shaking for 2 h. Two 15-mL aliquots were harvested at 1-h intervals, filtered through sterile gauze to remove undigested tissue, and immediately neutralized with an equal volume of prewarmed proliferation medium containing 20% FBS. Cell suspensions were centrifuged at 200 × *g* for 10 min at 4 °C. Resulting pellets were gently resuspended in fresh, prewarmed proliferation medium.

Cell viability and concentration were evaluated using 0.4% Trypan Blue dye exclusion and a Neubauer hemocytometer under a Nikon Eclipse E200 microscope (Nikon, Tokyo, Japan). Cells were seeded at a density of 3.5 × 10^5^ cells per well, in triplicate, on 12-well collagen I-coated plates containing 500 μL of proliferation medium per well. Cultures were incubated at 37 °C in a humidified atmosphere with 5% CO_2_, and half of the medium was replaced every 48–72 h to maintain optimal nutrient supply and remove metabolic byproducts. Morphology scoring was conducted by a blinded observer across ten non-overlapping 10× fields per donor. Cells showing rounded, blebbed, or highly vacuolated cytoplasm with detachment were counted as overtly injured; the proportion exceeded 10% in none of the fields.

### Proliferation kinetics and growth curve

Proliferation kinetics were determined by seeding 1 × 10^5^ GECs per well on collagen-coated 12-well plates with 500 µL of proliferation medium. Cells were incubated at 37 °C under 5% CO_2_ for a total of 5 days. Daily cell counts were performed every 24 h using Trypan Blue exclusion and a Neubauer chamber.

For quantitative growth curve analysis, 1 × 10^4^ cells per well were seeded in collagen-coated 96-well plates (total volume 200 µL). At predefined time points (24, 48, 72, 96, and 120 h), 10 µL of WST-1 reagent (Sigma-Aldrich, St. Louis, MO, USA) was added and incubated for 2 h at 37 °C. Absorbance was subsequently measured at 540 nm using an iMark™ microplate reader (Bio-Rad, Hercules, CA, USA), providing a robust correlation with metabolic activity. Preliminary calibration confirmed linearity versus direct cell counts under these settings.

### Hematoxylin and eosin (H&E) staining

A total of 1 × 10^4^ GECs were seeded onto sterile glass slides and cultured for 24 h to allow adequate adherence. Cells were then fixed with 4% paraformaldehyde (PFA) for 15 min at room temperature, stained with Mayer’s hematoxylin and 1% eosin Y solution (both from Merck, Darmstadt, Germany) for 30 s each, and rinsed with PBS to remove excess dye. AGS (CRL-1739) and 293T/17 (CRL-11268) cell lines (both from ATCC, Manassas, VA, USA) were employed as positive and negative controls, respectively. Prepared slides were examined under a Nikon Eclipse E200 microscope (Nikon, Tokyo, Japan) to assess general cellular morphology and confirm epithelial features.

### Immunofluorescence detection of cytokeratin

To verify epithelial lineage, 4 × 10^4^ GECs were seeded on collagen-coated coverslips and cultured for 24 h. Cells were fixed in 4% paraformaldehyde (Sigma-Aldrich), permeabilized with 0.02% Triton X-100 (Sigma-Aldrich), and blocked in 5% BSA + 2% normal sheep serum. Samples were incubated overnight at 4 °C with anti-cytokeratin 18 (mouse monoclonal; Santa Cruz Biotechnology; cat. no. sc-51582; RRID: AB_626608; lot no. B0604; 1:100), followed by Alexa Fluor 488 goat anti-mouse immunoglobulin G (IgG) (Thermo Fisher Scientific; cat. no. A-11001; lot no. 29,634; 1:500). Nuclei were counterstained with 4′,6-diamidino-2-phenylindole (DAPI) and slides mounted in UltraCruz^®^ mounting medium (Santa Cruz). Specificity controls comprised secondary-only (no-primary) and isotype-matched controls acquired under identical settings; both showed no specific signal. As biological controls, AGS (epithelial) and 293 T/17 (nongingival, nonkeratinocyte) cell lines were processed in parallel. Images were acquired on an EVOS FL Auto 2 fluorescence microscope (Thermo Fisher Scientific) using uniform exposure parameters across donors.

Quantification and scoring. For each donor and condition, ten non-overlapping fields at 10× were imaged under identical exposure. Images were analyzed in FIJI/ImageJ version 1.53 by an investigator blinded to group assignment. Background was estimated per field and subtracted from cytoplasmic signal. A cell was scored CK18-positive when its mean cytoplasmic fluorescence exceeded the field background mean by ≥ 2 standard deviation (SD); percentages of CK18-positive cells were computed per field and then averaged at the donor level for statistical analysis.

### Immunohistochemical detection of epithelial markers

GECs (4 × 10^4^ cells per well) were fixed and permeabilized with 0.1% Triton X-100, then blocked in 2% BSA. Samples were incubated overnight at 4 °C with mouse monoclonal antibodies against: MUC1 (Santa Cruz Biotechnology; cat. no. sc-7313; RRID: AB_626983; lot no. K2403; 1:100), pan-cytokeratin AE1/AE3 (Santa Cruz; cat. no. sc-81714; RRID: AB_2191222; lot no. B1302; 1:100), and vimentin (Santa Cruz; cat. no. sc-6260; RRID: AB_628437; lot no. G0692; 1:100). Detection used horseradish peroxidase (HRP)-conjugated goat anti-mouse IgG (Vector Laboratories; cat. no. MP-7452; lot no. 29,502), Vectastain ABC, and 3,3′-diaminobenzidine (DAB) chromogen, with hematoxylin counterstain (1 min). Isotype-matched and secondary-only controls were included to assess nonspecific binding; AGS and 293T/17 served as epithelial and nonepithelial biological controls, respectively. Bright-field images were obtained with a Nikon Eclipse E200 microscope.

Evaluation criteria. Immunohistochemistry (IHC) outcomes were assessed for expected subcellular localization and presence/absence of signal in defined compartments. A cell was considered positive when the DAB optical density in the expected compartment exceeded the field background mean by ≥ 2 SD. Accordingly, AE1/AE3 was recorded as diffuse cytoplasmic staining in polygonal epithelial cells; MUC1 as apical/supranuclear or membranous staining; and vimentin as cytoplasmic filamentous staining (negative in GECs and positive in 293T/17). When percentages are reported, the fraction of positive cells was calculated per field and averaged at the donor level; no *H*-score was computed.

### Antibody validation and specificity controls

Primary antibodies were validated in accordance with the journal’s *Policy on Antibody Validation*. For each target, the antibody source and catalog number are reported in the corresponding “Methods” subsections. Specificity was supported by (i) biological controls processed in parallel—AGS cells as epithelial positives and 293T/17 cells as nonepithelial, vimentin-positive controls—and (ii) technical controls—secondary-only (no-primary) and isotype-matched controls at identical concentrations. Acquisition settings were matched across samples. Positive controls exhibited the expected staining patterns, whereas technical controls showed no specific signal. For transparency, clone/host, lot number, and working dilution for each antibody are provided inline within the “Immunofluorescence/Immunohistochemistry Methods” where the antibodies are first described.

### RT-qPCR for epithelial gene expression

Total RNA was extracted from GECs at 0, 24, 48, 72, 96, and 120 h using TRIzol™ reagent (Invitrogen, Thermo Fisher Scientific, Waltham, MA, USA) following the manufacturer’s protocol. Briefly, 1 mL of TRIzol™ was added to each cell pellet, homogenized thoroughly, and incubated at room temperature for 5 min. Chloroform (200 µL) was added, vortexed vigorously for 15 s, incubated for 3 min, and centrifuged at 12,000 × *g* for 15 min at 4 °C. The aqueous phase was collected, mixed with isopropanol (500 µL), incubated at −20 °C for 10 min, and centrifuged at 12,000 × *g* for 10 min. The resulting RNA pellet was washed with 75% ethanol, air-dried briefly, and resuspended in 30 µL of RNase-free water.

Concentration and purity were assessed by using a NanoDrop™ 2000 spectrophotometer (Thermo Fisher Scientific), accepting A260/A280 ratios of 1.8–2.0. For complementary DNA (cDNA) synthesis, 1 µg of RNA per sample was used with the High-Capacity cDNA Reverse Transcription Kit (Applied Biosystems, Foster City, CA, USA). The reaction (20 µL) included random primers, deoxynucleotide triphosphates (dNTPs), reverse transcriptase, and buffer, with thermal cycling at 25 °C for 10 min, 37 °C for 120 min, and 85 °C for 5 min, then held at 4 °C.

Quantitative polymerase chain reaction (PCR) was performed using TaqMan™ Universal PCR Master Mix and TaqMan™ Gene Expression Assays for *MUC1* (Hs00159357_m1), *MUC5AC* (Hs01365616_m1), and *GAPDH* (Hs02758991_g1) as the endogenous control. Each 20 µL reaction contained 10 µL of Master Mix, 1 µL of assay, 2 µL of cDNA, and 7 µL of RNase-free water.

Reactions were run on a StepOnePlus™ Real-Time PCR System (Applied Biosystems) under the following conditions: 95 °C for 10 min, then 40 cycles of 95 °C for 15 s and 60 °C for 1 min. Samples were analyzed in triplicate to ensure consistency. Data are summarized as ΔCt (Ct_gene − Ct_*GAPDH*). No calibrator was applied; lower ΔCt indicates higher expression. Ct values above 35 were considered negligible. No-template controls and RT-minus controls were included to exclude contamination and genomic DNA amplification. All reagents and plastics were RNase-free to preserve RNA integrity.

### Mycoplasma testing

All cultures were screened for mycoplasma contamination using the MycoAlert™ Detection Kit (Lonza, Basel, Switzerland) according to the manufacturer’s instructions. All samples consistently tested negative, confirming the absence of contamination.

### Microscopy and image acquisition/processing

Bright-field and phase-contrast micrographs were acquired on a Nikon Eclipse E200 upright microscope (Nikon, Tokyo, Japan) equipped with a Plan Achromat 10×/0.25 NA objective and a Ph1 phase condenser, using identical optical settings across donors. Fluorescence images were captured on an EVOS FL Auto 2 system (Thermo Fisher Scientific) fitted with light-emitting diode (LED) light cubes for DAPI (nuclear), GFP/Alexa Fluor 488, and RFP/Alexa Fluor 594/568 channels (bandpass filter sets as supplied by the manufacturer). Images were recorded with the EVOS integrated monochrome camera at its native sensor resolution (2048 × 1536 pixels). Exposure times were 8–15 ms for bright-field/phase-contrast and 80–250 ms for fluorescence; detector gain was 0–1 (factory default); binning was 1 × 1; gamma was off. Stage-micrometer calibration yielded an *x*–*y* sampling of 0.65 µm/pixel at 10×; linear scaling the 4× objective provided 1.63 µm/pixel. Unless otherwise stated, images were acquired at 10×; only the overview immunofluorescence panels shown in Fig. 3 (top row) were captured at 4× to visualize the colony-wide CK18 distribution. No *z*-stacks or time series were acquired (*z* = 0; *t* = 0). The bit depth was 8 bits (bright-field/phase-contrast) and 16 bits (fluorescence). Scale bars were generated in FIJI from the above calibrations and embedded in the composites: 200 µm for the 4× immunofluorescence panels and 100 µm for 10× bright-field/IHC panels.

### Statistical analysis

Analyses were performed at the donor level (*n* = 3), averaging technical replicates within each donor before testing. Data are reported as mean ± SD; normality was assessed with the Shapiro–Wilk test. Time-course outcomes (direct cell counts and WST-1 absorbance) were analyzed with one-way repeated-measures analysis of variance (ANOVA) using the Geisser–Greenhouse correction, followed by Tukey’s multiple comparisons test (*α* = 0.05). Population doubling time (DT) was calculated as DT = *t* × log10(2)/[log10(N2) − log10(N1)], where N1 and N2 are the cell numbers at the beginning and end of interval *t*. RT-qPCR results are summarized as ΔCt (Ct_gene − Ct_*GAPDH*); Ct > 35 was considered below the reliable quantification limit and was not used for inference. Pearson’s correlation (*r*) was computed between donor-level means of WST-1 absorbance and direct cell counts across time points. Analyses were run in GraphPad Prism version 9.0 (GraphPad Software, San Diego, CA, USA).

## Ethics statement

This study was conducted in strict accordance with the Declaration of Helsinki (2013 revision), ensuring respect for human dignity, autonomy, and rights throughout all procedures. The protocol was thoroughly reviewed and approved by the Research Ethics Committee of the Universidad de Antioquia (approval act no. 012–2022). All participants received comprehensive verbal and written information regarding the objectives, procedures, potential risks, and benefits of the study, and provided written informed consent before any tissue collection. Furthermore, the research fully adhered to the ethical guidelines established under Colombian law, specifically Resolution No. 8430 of 1993 issued by the Ministry of Health, which regulates health research involving human subjects. According to this resolution, the study was classified as minimal risk, as it involved collection of gingival tissue from healthy volunteers undergoing elective surgical procedures without additional intervention. All donor data were anonymized to ensure confidentiality and were handled in accordance with national and institutional data protection policies.

## Results

### Early morphological characterization and expansion dynamics of primary gingival epithelial cells

Following mechanical–enzymatic dissociation, GECs were seeded onto collagen-coated plates to promote rapid and stable attachment. The average yield was (1.02 ± 0.18) × 10^6^ viable cells per gram of tissue, with viability consistently above 92% (*n* = 3). This outcome reflected efficient dissociation with limited overt injury, defined as: (i) Trypan Blue viability ≥ 90% immediately post-isolation; (ii) absence of widespread apoptotic morphology (membrane blebbing, nuclear condensation) in > 90% of cells across ten random fields; and (iii) preservation of an epithelial marker profile (CK18/AE1-AE3 positive, vimentin negative) within 24–48 h. Quantitative viability and morphology scoring criteria are provided in “Methods.”

**Fig. 1:**
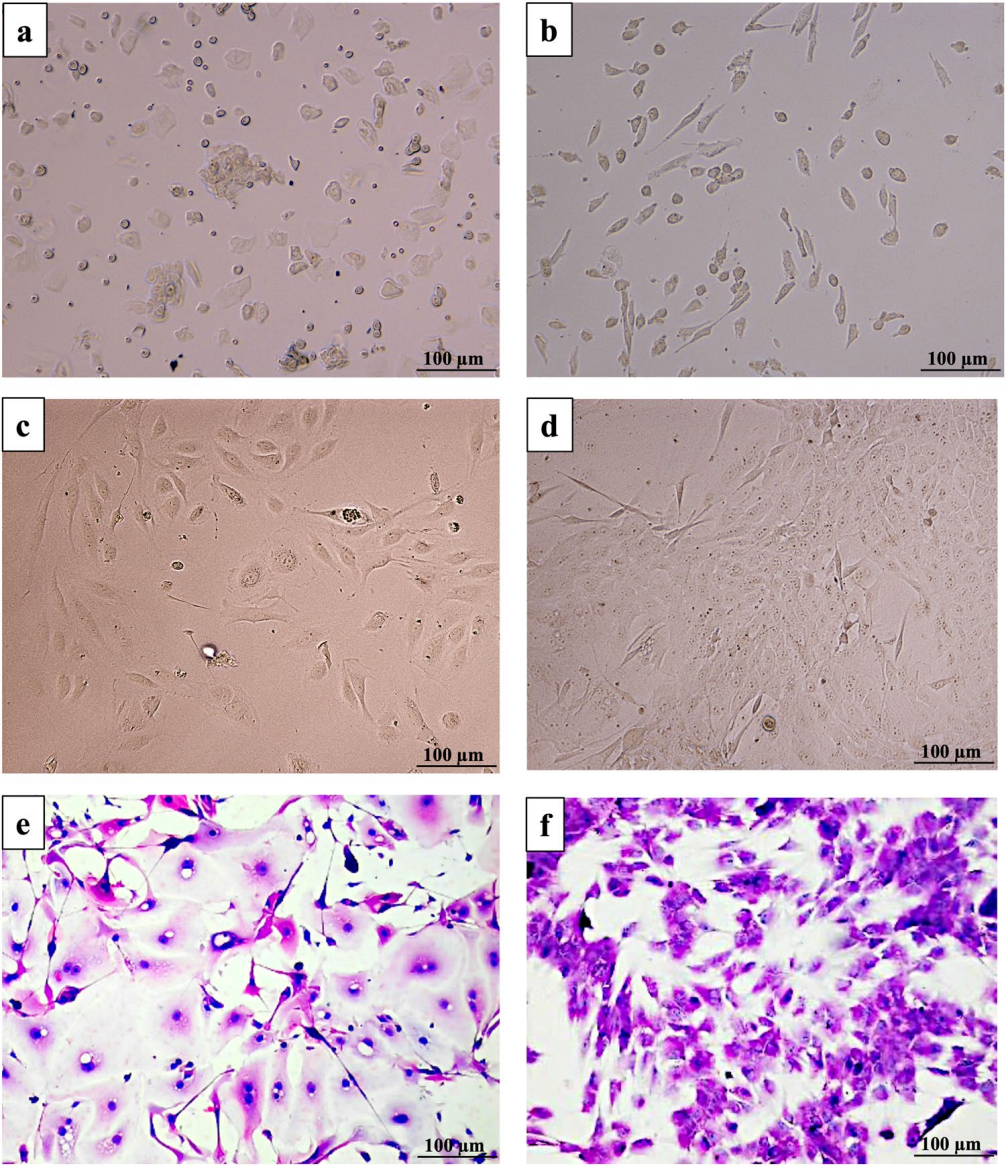
Morphological progression of gingival epithelial cells (GECs) during early culture: phase-contrast and hematoxylin–eosin micrographs. Phase-contrast images (**a**–**d**) illustrate the sequential morphological changes of GECs following enzymatic isolation and seeding on type I collagen. Panel **a** shows cells at day 0 (immediately after seeding), panel **b** at 24 h, panel **c** at 72 h, and panel **d** at day 5. A progressive pattern of attachment, spreading, and expansion is observed, with cells transitioning from a rounded to a flattened, polygonal morphology characteristic of epithelial phenotype. Panel **e** shows hematoxylin–eosin staining of GECs at day 5, confirming polygonal architecture, prominent nuclei, and distinct intercellular borders. Panel **f** corresponds to the nonepithelial control (293T/17 cells), stained with hematoxylin–eosin, showing a fibroblastoid, mesenchymal-like morphology with spindle-shaped cells and disorganized growth. Images are representative of three independent donors. Scale bars: 100 µm

By 24 h, phase-contrast images showed early adhesion with discrete focal contacts and spreading across the substrate (Fig. 1b). At 48 h, cells adopted a cohesive polygonal shape with central nuclei, high nuclear-to-cytoplasmic ratios, and sharply defined intercellular borders, consistent with a basal epithelial phenotype (Fig. 1c).

Between 48 and 72 h, radial outgrowth advanced uniformly. At the colony edge, cells extended short lamellipodia, indicative of coordinated sheet migration rather than fibroblastic drift (Fig. 1c–d). Occasional perinuclear vacuoles were observed in a minority of cells (< 10%), interpreted as Golgi/secretory vesicles in metabolically active keratinocytes rather than degenerative changes. These features decreased in frequency as cultures approached confluence (Fig. 1b, c).

By day 5, monolayers reached ~ 90% confluence, preserving epithelial polarity and uniform architecture without multilayering or overlap. No fibroblastic contamination or signs of epithelial–mesenchymal transition were detected. Hematoxylin and eosin staining confirmed cohesive epithelial sheets with prominent nuclei and well-defined junctions (Fig. 1e). In contrast, the nonepithelial control line 293T/17 displayed elongated, disorganized morphology with indistinct borders and reduced intercellular contacts (Fig. 1f).

Collectively, these observations validate the structural integrity and epithelial identity of primary GEC cultures during early expansion, supporting their suitability for downstream functional and molecular applications.

### Proliferation kinetics and metabolic activity of primary gingival epithelial cells

The proliferative dynamics and metabolic activity of primary GECs were assessed through complementary approaches: manual cell counts using Trypan Blue exclusion and mitochondrial metabolic evaluation via WST-1 assays. These analyses were performed at sequential intervals to thoroughly describe early expansion behavior under defined epithelial culture conditions.

**Fig. 2:**
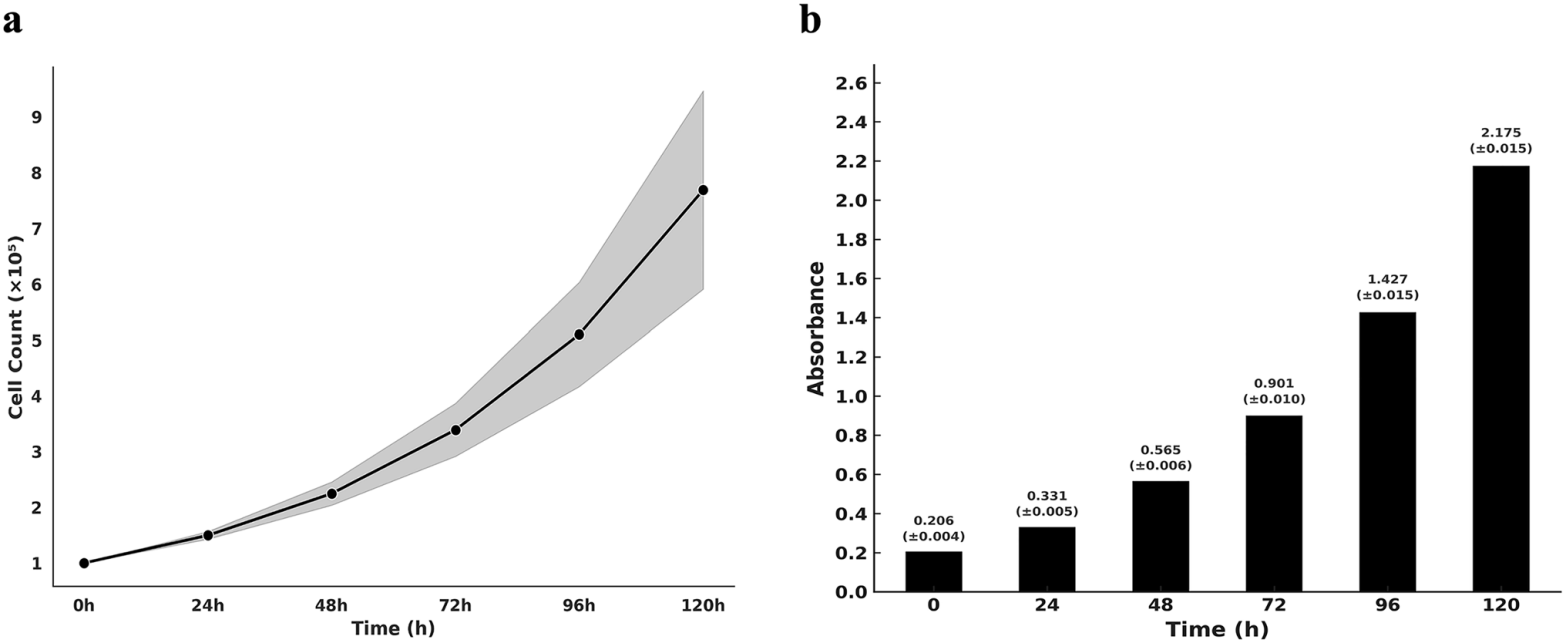
Proliferation kinetics and metabolic activity of primary human gingival epithelial cells (GECs) **a** A progressive increase in cell number was observed throughout the 120-h culture period, with a well-defined exponential growth phase occurring between 48 and 96 h. This dynamic reflects active cell division and efficient adaptation to the in vitro environment. The calculated average population doubling time was 41.6 ± 3.8 h, demonstrating stable proliferative capacity under standardized conditions and minimal donor variability. **b** In parallel, WST-1-based quantification of mitochondrial dehydrogenase activity showed a gradual rise in absorbance at 540 nm across all time points, supporting a sustained increase in metabolic output. The concordance between proliferative expansion and metabolic activity suggests a preserved epithelial phenotype with high bioenergetic efficiency. Data represent mean ± SD from three donors

GECs demonstrated a clear biphasic growth pattern, with an initial lag phase during the first 24 h characterized by minimal net increase in cell number, likely reflecting substrate adaptation and cytoskeletal reorganization. This phase was followed by a pronounced exponential expansion between 48 and 96 h, corresponding to active cell cycle progression and collective sheet migration. By day 5, cultures consistently achieved near-confluence, indicating robust proliferative potential and high plating efficiency. The calculated average doubling time was 41.6 ± 3.8 h, with low interdonor variability, confirming interdonor consistency of the isolation and culture protocol. Viability, assessed in parallel, remained above 90% throughout the 5-day window, confirming preservation of cellular integrity and absence of significant apoptosis or detachment events (Fig. 2a).

Metabolic activity measurements corroborated these findings. The WST-1 assay revealed a steady and statistically significant increase in absorbance at 540 nm across all evaluated time points, reflecting enhanced mitochondrial dehydrogenase activity and sustained bioenergetic output as cultures progressed toward confluence. Mean ± SD absorbance values were 0.21 ± 0.004 at baseline (0 h), 0.33 ± 0.005 at 24 h, 0.57 ± 0.006 at 48 h, 0.90 ± 0.010 at 72 h, 1.43 ± 0.015 at 96 h, and 1.95 ± 0.010 at 120 h. One-way ANOVA with Tukey’s post hoc analysis confirmed a significant temporal increase (p < 0.01), validating the assay’s sensitivity and linear performance under standardized conditions across donors.

Furthermore, a strong positive correlation was established between WST-1 absorbance values and direct cell counts (Pearson’s *r* = 0.994, *p* = 5.6 × 10^−5^), indicating that WST-1 can reliably serve as a surrogate metric for cell proliferation monitoring in primary GEC systems (Fig. 2b). This correlation reinforces the methodological robustness and supports future application of metabolic assays for noninvasive tracking of growth kinetics in similar epithelial models.

Collectively, these results demonstrate that primary gingival epithelial cells exhibit consistent proliferative capacity and sustained metabolic activity during early in vitro expansion. The integration of morphological, numerical, and metabolic data objectively characterizes early culture behavior and confirms interdonor consistency of the isolation and culture workflow under standardized within-laboratory conditions.

### Phenotypic and transcriptomic validation of gingival epithelial cell identity

**Fig. 3:**
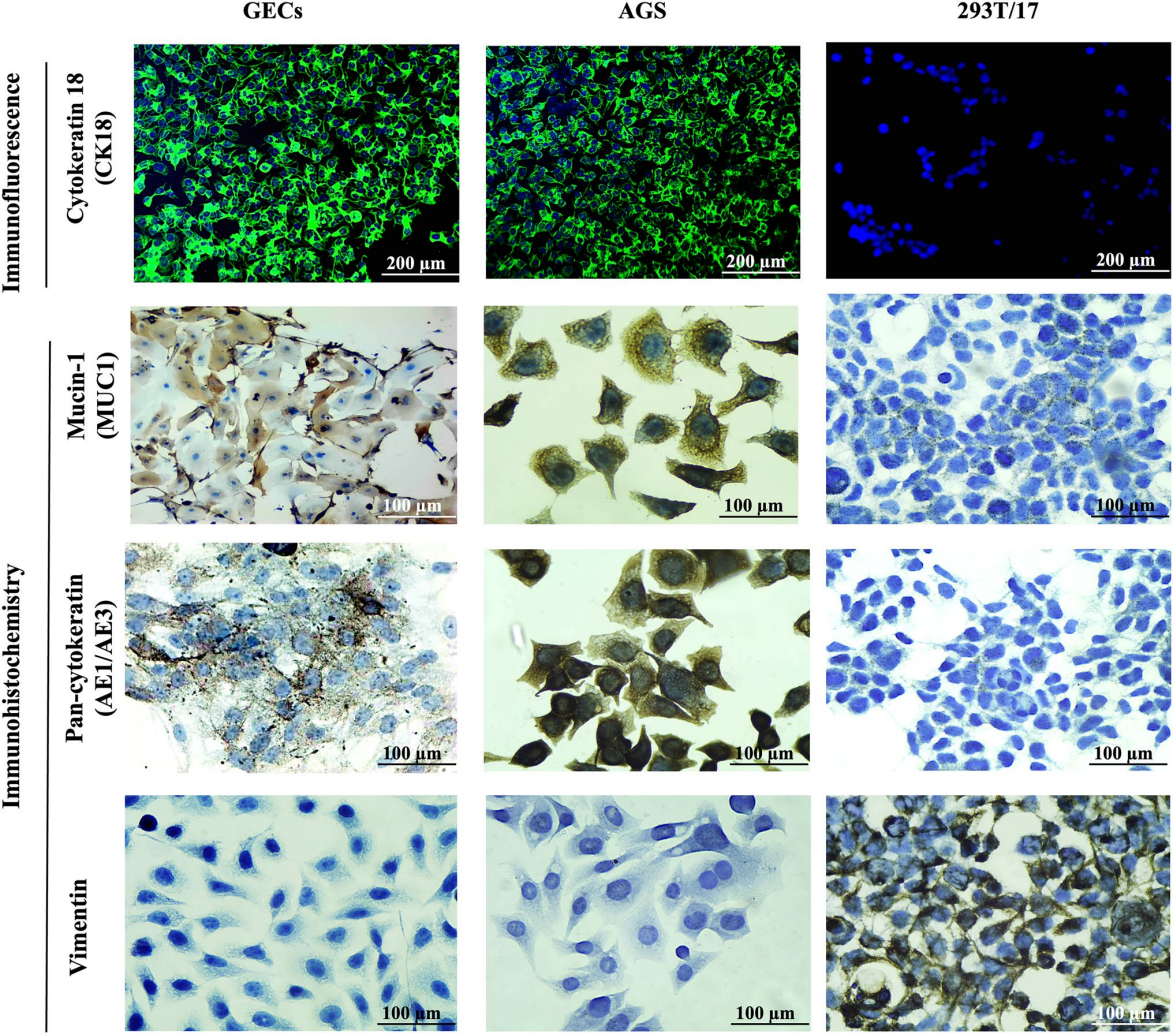
Expression of epithelial and mesenchymal markers in primary gingival epithelial cells. Representative immunofluorescence and immunohistochemical staining of GECs, AGS (epithelial control), and 293T/17 (nonepithelial control). Top row: CK18 immunofluorescence shows strong perinuclear staining in GECs and AGS (green; Alexa Fluor 488). Nuclei are counterstained with DAPI (blue). No CK18 signal is observed in 293T/17. Second row: MUC1 exhibits apical and supranuclear localization in GECs and AGS, absent in 293T/17. Third row: AE1/AE3 pan-cytokeratin staining is evident in GECs and AGS, confirming epithelial phenotype; 293T/17 are negative. Bottom row: vimentin expression is restricted to 293T/17, with complete absence in epithelial cultures. Representative images from three independent donors. Scale bars: 200 µm (top-row immunofluorescence); 100 µm (rows 2–4, bright-field/IHC)

The epithelial origin and keratinocyte phenotype of GECs were rigorously confirmed through a multimodal approach integrating immunofluorescence, immunohistochemistry, and RT-qPCR analysis. Immunofluorescence staining demonstrated robust cytoplasmic expression of cytokeratin 18 (CK18), a type I intermediate filament indicative of simple and nonkeratinized stratified epithelia. A distinct perinuclear filamentous distribution was evident in 96.4 ± 2.2% of cells across all examined fields (*n* = 12 per donor, three donors). CK18 was completely absent in nonepithelial 293T/17 controls and distinctly present in AGS epithelial cells, confirming both assay specificity and methodological consistency (Fig. 3, top row).

Supporting these findings, immunohistochemistry revealed MUC1 expression in 78.6 ± 3.2% of GECs, displaying an apical and granular perinuclear staining pattern consistent with epithelial differentiation (Fig. 3, second row). The pan-cytokeratin marker AE1/AE3 labeled 97.2 ± 1.1% of cells, exhibiting uniform cytoplasmic staining that reinforced epithelial integrity (Fig. 3, third row). Vimentin expression was undetectable in GECs but strongly positive in 293T/17 cells, which also lacked CK18, MUC1, and AE1/AE3, while AGS controls recapitulated the GEC profile and were uniformly vimentin-negative (Fig. 3, bottom row).

**Fig. 4:**
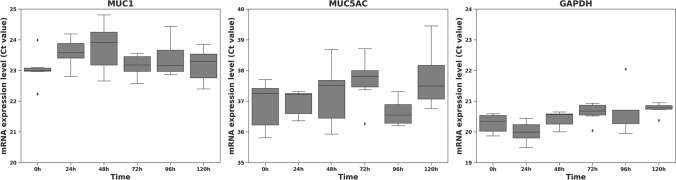
RT-qPCR Ct values for *MUC1*, *MUC5AC*, and *GAPDH* in primary gingival epithelial cells. Raw Ct values (donor-level means; technical replicates averaged within donor) are shown at 0, 24, 48, 72, 96, and 120 h. Boxes indicate the median and interquartile range, and whiskers the minimum–maximum (*n* = 3 donors). *MUC1* remained stable over time (Ct ≈ 23), consistent with preserved epithelial identity. *MUC5AC* was undetected or above the reliable quantification limit (Ct > 35) at all time points. *GAPDH* served as the reference gene and was stable (Ct ≈ 20–21). Statistical analyses were performed on ΔCt (see “Methods”)

At the transcript level, ΔCt analysis showed that *MUC1* expression remained stable from baseline to 120 h: ΔCt (*MUC1 − GAPDH*) fluctuated only minimally around the starting value with no time-dependent trend across donors. In contrast, *MUC5AC* stayed below the reliable quantification limit (Ct > 35 or undetected at all time points), indicating negligible expression. *GAPDH* Ct values were tightly clustered (20.1–20.8), supporting RNA quality and assay reproducibility (Fig. 4).

Altogether, the consistent expression of CK18, AE1/AE3, and MUC1, combined with the absence of vimentin and MUC5AC, underscores the lineage fidelity and phenotypic stability of these primary cultures. This comprehensive validation supports the suitability of the model for advanced investigations in oral epithelial biology, including barrier function, regenerative strategies, and host–microbe interaction studies.

Moreover, GEC cultures were maintained for up to 14 days without evidence of morphological alterations or loss of adhesion. Throughout this period, cells preserved their polygonal architecture and monolayer cohesion, with no signs of apoptosis, detachment, or keratinized differentiation, as verified by serial inspections using an inverted phase-contrast microscope. These additional observations, although not shown, further emphasize the robust stability and viability of this primary epithelial model.

## Discussion

Gingival epithelial cells (GECs) represent a physiologically relevant in vitro platform to investigate host–microbe interactions, epithelial regeneration, and periodontal pathology. Unlike immortalized keratinocyte lines such as OKF6 and HaCaT, which often display phenotypic drift and altered signaling cascades (Chapman et al. 2010), primary GECs maintain authentic transcriptional profiles and native responsiveness to stimuli (Groeger and Meyle 2019). Nevertheless, establishing pure, stable cultures is challenging owing to donor variability, limited epithelial yield, and risks of fibroblast overgrowth or mesenchymal transition (Piwocka et al. 2024; Müssig et al. 2008). The combined mechanical–enzymatic dissociation protocol described here enhances early epithelial adhesion and effectively minimizes nonepithelial contamination, improving lineage fidelity and experimental reproducibility. This methodological advancement strengthens its applicability for studies in mucosal immunology, barrier function analysis, and peri-implant tissue research.

The morphology documented in cultured GECs closely mirrored the features of basal keratinocytes, providing robust evidence of their epithelial origin. Characteristically polygonal shape, intermediate cell size, and a pronounced nuclear-to-cytoplasmic ratio are hallmarks of stable and healthy epithelial monolayers (Angelova Volponi et al. 2013; Neunzehn et al. 2012). The consistent presence of these morphological attributes across all donor-derived cultures indicates that the current protocol effectively limits donor-dependent variability and supports early phenotypic stabilization, a critical factor for reproducibility in translational studies. Moreover, the clear absence of spindle-like cells, cytoplasmic extensions, or irregular protrusions strongly argues against mesenchymal contamination or spontaneous epithelial–mesenchymal transition, complications frequently encountered in primary keratinocyte isolations (Xie et al. 2021; Giannobile et al. 1998). Comparative analysis using 293T/17 and AGS cell lines as negative and positive controls, respectively, provided further validation; both controls exhibited distinct and reproducible morphologies, corroborating the specific epithelial architecture observed in GECs. Such morphological fidelity is essential for downstream functional assays, including migration, wound closure, and barrier integrity evaluations. Adherence to these stringent morphological benchmarks aligns with established cytological standards for assessing epithelial keratinocyte purity and culture quality (Sztukowska et al. 2016), ensuring that the resulting monolayers remain a reliable foundation for mechanistic and regenerative investigations.

The proliferation dynamics observed in primary GEC cultures demonstrated a biphasic growth pattern that is characteristic of freshly isolated epithelial cells. Following an initial adaptation phase, cells entered exponential growth between 48 and 96 h, ultimately reaching near-confluence by day 5. This temporal profile aligns with observations from Kedjarune et al., who emphasized the importance of substrate interaction and early adaptation for optimal proliferation rates in gingival keratinocytes (Kedjarune et al. 2001). The calculated doubling time of 41.6 ± 3.8 h further confirms preserved mitotic competence and viability, consistent with data from organotypic mucosal models that rely on primary cells to maintain physiological relevance (Klausner et al. 2021). Additionally, the viability consistently above 90% throughout the expansion period underscores the effectiveness of the culture environment and the absence of significant cytotoxic stress, factors critical for maintaining epithelial functionality. The strong correlation between WST-1 metabolic activity and direct cell counts validates the use of metabolic assays as reliable surrogates for proliferation monitoring in GECs. This metabolic resilience reflects intact mitochondrial functionality, a prerequisite for supporting sustained epithelial barrier function and regenerative potential (Sakamoto et al. 2015). Notably, Ortiz-Arrabal et al. highlighted that robust mitochondrial activity is essential to uphold not only cellular energetics but also the secretory and defensive roles of oral epithelia (Ortiz-Arrabal et al. 2022). By integrating proliferation metrics with metabolic profiling, this study offers a comprehensive characterization framework that can inform future applications in regenerative dentistry, implantology, and mucosal infection models.

The immunophenotypic and transcriptomic characterization further consolidates the epithelial identity and stability of the GEC cultures. Cytokeratin 18, expressed in more than 96% of cells, displayed a prominent perinuclear filamentous staining pattern, which is a defining marker of nonkeratinized, simple stratified epithelial cells (Hormia et al. 1987; Nanda et al. 2012). This distribution pattern corroborates findings in native human gingival tissue and supports the maintenance of epithelial integrity during in vitro expansion. Additionally, the co-expression of the pan-cytokeratin marker AE1/AE3 in over 97% of cells, with diffuse cytoplasmic localization, mirrors data from Clausen et al. and offers further confirmation of epithelial phenotype preservation (Clausen et al. 1986). The complete absence of vimentin expression in GECs, in contrast to its robust presence in 293T/17 nongingival, nonkeratinocyte controls, excludes mesenchymal contamination and underscores the lineage specificity of the protocol. The detection of MUC1 in approximately 80% of cells, with a distinct apical and supranuclear distribution, highlights the preservation of epithelial polarity and suggests maintained mucosal barrier-associated functionality (Hansson et al. 2001; Sirviö et al. 2019; Ukkonen et al. 2017). Conversely, MUC5AC, typically indicative of glandular or metaplastic differentiation, remained undetectable or at negligible levels across all time points, indicating no drift toward a glandular phenotype (Lin et al. 2020). The stable Ct values for *GAPDH* across all samples ensured consistent RNA integrity and assay reliability, providing confidence in transcriptomic conclusions. Collectively, this integrated immunohistochemical and gene expression profile confirms the phenotypic stability, epithelial purity, and functional potential of the GECs, supporting their applicability in studies investigating mucosal immunity, wound repair mechanisms, and epithelial–microbial interactions.

In comparison with commercially available 3D gingival constructs such as EpiOral™ and SkinEthic™ HOE, the donor-derived GEC monolayers presented here prioritize phenotypic fidelity, donor resolution, and scalability. While many multilayer systems still rely on immortalized keratinocyte lines (e.g., TR146) with documented alterations in differentiation programs, signaling pathways, and transcriptional profiles (AlFatlawi et al. 2023; Golda et al. 2023), primary-cell-based gingival equivalents have also been established and shown to preserve donor-specific features (Plaza et al. 2024; Zhang et al. 2022). Our approach is complementary to these primary-cell 3D models: a feeder-free, rigorously validated monolayer that can *seed* donor-resolved 3D reconstructions or co-culture challenges under defined conditions. In practice, generating multilayer constructs—even with primary cells—often requires complex scaffolds, extended culture periods, and higher costs, which can limit throughput and patient-specific experimentation (Colley et al. 2013). By contrast, our protocol yields robust monolayers without artificial matrices or prolonged conditioning, and their morphology and molecular signatures align closely with in vivo gingival epithelia (Ukkonen et al. 2017; Lamont et al. 2018). This combination of fidelity, scalability, and donor specificity enables translational applications—including antimicrobial testing, evaluation of regenerative biomaterials, and disease modeling at the epithelial barrier—and helps bridge preclinical in vitro work with clinically relevant questions in oral medicine (Dickinson et al. 2011; Clausen et al. 1986).

Despite the evident strengths of this model—such as phenotypic stability, epithelial fidelity, and high within-laboratory interdonor consistency—it is important to acknowledge its limitations. The current study assessed cultures up to 5 days, providing detailed insights into early proliferation, morphology, and marker expression; however, longer-term maintenance, including serial passaging and behavior beyond 2 weeks, remains unexplored. Future investigations should address whether these primary GECs preserve their epithelial characteristics, barrier integrity, and functional responses under chronic exposure to inflammatory mediators, microbial pathogens, or mechanical stimuli. Such extended studies would help elucidate their suitability for applications in long-term regenerative assays, host–microbe interaction modeling, and drug screening platforms. Moreover, the sample size was limited to three donors, which restricts generalizability and precludes comprehensive evaluation of interindividual variability related to genetic background, age, or oral health status. Expanding donor cohorts and including diseased tissue sources could further strengthen the translational relevance and facilitate personalized medicine approaches in periodontal and mucosal research. Nonetheless, the present work establishes a phenotypically faithful platform with demonstrated interdonor consistency within a young-adult cohort, laying the foundation for future mechanistic and therapeutic studies. As such, it offers a valuable resource for advancing our understanding of oral epithelial biology and supports translational efforts aiming to develop targeted therapies and regenerative strategies for periodontal and peri-implant conditions.

## Data Availability

All datasets generated and/or analyzed in this study—including raw cell-proliferation counts, WST-1 readouts, microscopy images, and RT-qPCR data—will be made available by the corresponding author upon reasonable request.

## Declarations

### Open Access

This article will be published under the standard terms of the publisher's open access policy if accepted.
